# Effects of long-term afforestation on soil water and carbon in the Alxa Plateau

**DOI:** 10.3389/fpls.2023.1273108

**Published:** 2024-01-11

**Authors:** Xinglin Zhu, Jianhua Si, Xiaohui He, Bing Jia, Dongmeng Zhou, Chunlin Wang, Jie Qin, Zijin Liu

**Affiliations:** ^1^ Key Laboratory of Eco-Hydrology of Inland River Basin, Northwest Institute of Eco-Environment and Resources, Chinese Academy of Sciences, Lanzhou, China; ^2^ University of Chinese Academy of Sciences, Beijing, China; ^3^ Faculty of Resources and Environment, Baotou Teachers’ College, Inner Mongolia University of Science and Technology, Baotou, China

**Keywords:** soil organic carbon, soil inorganic carbon, soil water, afforestation, stand

## Abstract

Plantations in dry and semi-arid areas significantly affect the soil’s ability to store carbon and maintain a stable water balance. It is yet unclear, though, how planted trees in these regions might impact the soil’s carbon and water levels. As a forest ages, it is unknown how soil water and soil carbon interact with one another. In order to conduct this study, four Saxaul plantations in the Alxa Plateau were chosen, with the neighboring mobile sandy (MS) ground serving as a control. The ages of the plantations ranged from 5 to 46 years. The major topics of the study included the relationship between soil water and soil carbon, changes in the 0-300 cm soil layer’s soil water content (SWC), soil organic carbon (SOC), and soil inorganic carbon (SIC) following afforestation. The findings demonstrated that, in comparison to MS, afforestation considerably increased SOC and SIC stocks. In comparison to MS, the SIC grew by 4.02 kg m^-2^, 4.12 kg m^-2^, 5.12 kg m^-2^, and 6.52 kg m^-2^ throughout periods of 5 years, 11 years, 22 years, and 46 years, respectively. SOC increased relative to MS by 2.55 kg m^-2^, 2.91 kg m^-2^, 3.53 kg m^-2^, and 4.05 kg m^-2^. Afforestation, however, also contributed to a considerable decline in deep SWC and an increase in the soil water deficit (SWD). In comparison to MS, the mean SWC values were lower at 5 years, 11 years, 22 years, and 46 years, respectively, by 0.48%, 1.37%, 1.56%, and 4.00%. The increase in soil carbon pool caused by sand afforestation actually came at the expense of a reduction in soil water due to a large negative association between deep SWC, SOC, and SIC. To limit SWC losses and encourage sustainable forest land development, we advocate suitable harvest management practices on forest land.

## 1 Introduction

Environmental concerns, such as climate change and land degradation, have long been of great importance. Afforestation has recently become recognized as an essential ecological strategy for soil restoration, halting land degradation, and reducing climate change (Lal, 2008; Deng et al., 2016a; Conant et al., 2017). According to numerous studies (Mobley et al., 2015; Wang et al., 2016; Zhao et al., 2016a), afforestation improves ecosystem resilience and encourages soil carbon sequestration in arid, semi-arid, and desertified regions. Afforestation, on the other hand, can result in substantial changes in land use, which can have a considerable impact on soil water and the carbon pool. Soil water balance and carbon dynamics must be carefully examined while the region is revegetating.

Previous studies have noted a number of advantages of afforestation, including improving the ecological environment, supporting soil quality restoration, and lowering soil erosion (Keesstra et al., 2009; Mohawesh et al., 2015). Afforestation, however, can also be detrimental to the local ecology. In particular, soil water content (SWC) may be declining after afforestation, and deeper soils may experience more severe soil water deficit (SWD) and soil drying (Yang et al., 2020). When afforestation density exceeds the limiting carrying capacity of soil water, deep soil water loss becomes severe (Jia et al., 2017). Nevertheless, as the age of the stand increased, the SWC varied along the profile and varied between species and sites. Deep SWC has been seen to drop with increasing stand age in apple and black locust plantations in China, for example, in regions with an average annual precipitation< 500 mm (Gao et al., 2018a; Liang et al., 2018). By compiling data from 5668 post-afforestation loess plateaus, (Li et al., 2021) discovered that SWC continued to decline in the early stage of afforestation (20 years) and remained largely stable in the late stage of afforestation (> 20 years). Deep SWC grew initially and then decreased with increasing stand age, according to (Liu et al., 2010). These contradictory results imply that SWC modifications following afforestation varied for various places and vegetation types.

Soil inorganic carbon (SIC) pools and soil organic carbon (SOC) pools are types of soil carbon pools. After afforestation, there are variations in the dynamics of soil carbon pools, which are similar to the changes in SWC. Numerous studies have shown that post-forestation stand age has a major impact on soil carbon reservoirs (Deng et al., 2014; Deng et al., 2016a). According to previous studies (Pregitzer and Euskirchen, 2004; Bradford and Kastendick, 2010), the age of the forest stand has been found to be a significant predictor of soil carbon reservoirs in forest ecosystems. Research conducted by (Mao et al., 2010) showed an increase in soil carbon storage with stand age. Conversely, (Olszewska and Smal, 2008) reported a decrease in soil carbon storage, while (Sartori et al., 2007) found no significant change. These findings were based on field research conducted on soil carbon storage after afforestation. These multiple research demonstrate that stand age-related variations in soil carbon storage are not constant across habitat types and vegetation types. As a result of afforestation, changes in soil carbon stores and SWC are not two separate processes, but rather a reciprocal feeding mechanism (Liu et al., 2010). It is well acknowledged that raising the soil carbon pool enhances soil structure, encourages precipitation infiltration, and helps soils retain water. The development of plants and soil carbon sequestration are both dependent on successful SWC consumption (O'Brien et al., 2010). When afforestation occurs in arid and semi-arid regions, it is unclear if there is a positive or negative feedback between the restricted soil water and soil carbon sequestration.

Alxa League is a typical dry and semi-arid region and is situated in northwest China’s interior. Alxa is one of the most prone areas in China to sandstorms because of its ongoing drought, lack of water, and limited vegetation. Extensive afforestation aiming for erosion control and sand fixing started in the 1950’s. To some extent, sand-fixing afforestation can be used to illustrate the usual traits of arid and semi-arid locations. Due to its tolerance to drought and aridity, Saxaul plantations, a genus of Quinoa, is known as “desert plantation” and is the most common sand-fixing tree in the desert regions of the Asian hinterland. The following three aspects were looked into in this study based on the selection of Saxaul plantations at various stages in typical areas: (1) the changes of soil organic and inorganic carbon with forest age after afforestation; (2) the changes of soil water with depth and forest age after afforestation; and (3) the reciprocal feedbacks mechanism between soil water and soil carbon pool. The purpose of this study is to understand more fully the changes in soil water and soil carbon pools after afforestation in arid and semi-arid areas and the reciprocal feedback between them.

## 2 Materials and methods

### 2.1 Site description

**Figure 1:**
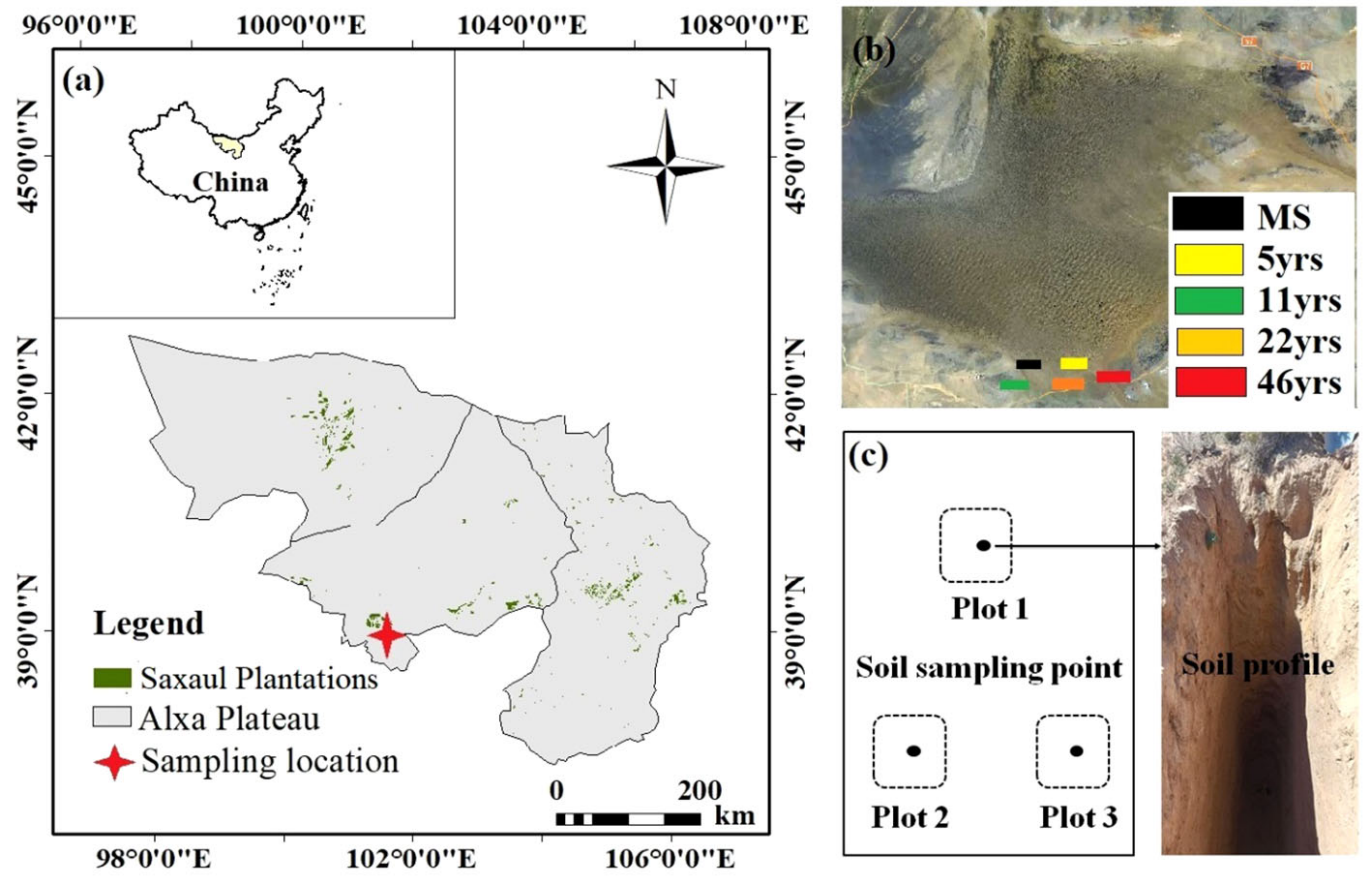
Location of the study area and soil sampling points. **(A)** Location of the study area, **(B)** Distribution of pike at different stand ages, and **(C)** Schematic illustration of soil sampling.

The Alxa Plateau is a region of around 270,000 km^2^ in the westernmost portion of the Inner Mongolia Autonomous Region. It is situated at 97°10′-106°53′E and 37°24′-42°47′N. With the Tengri, Badain Jaran, and Ulan Buh deserts, it is one of China’s locations that has been most severely desertified. With a 15% to 30% vegetation cover, the Alxa Plateau is geographically situated in the Alxa Desert Province, the easternmost subregion of the Asian desert flora in the Central Asian desert subregion (Gobi Desert subregion). High plains make up the majority of the region, followed by huge desert and Gobi regions, grassland regions, and minor mountain, woodland, and arable regions. Its elevation ranges from 820 to 1 400 meters. The terrain is typical of an arid desert, with arid weather and high winds. The general trend of precipitation distribution in Alxa League is more in the south and less in the north, more in the east and less in the west, decreasing from southeast to northwest. The distribution of precipitation is extremely uneven, primarily falling in the months of May and September, with the average annual precipitation ranging from 35.5 to 149.0 mm. The average annual temperature is 8.3°C, and the average annual evaporation is from 2400 to 4200 mm. Weather information above is provided by the meteorological service of Alxa League. From northwest to southeast, gray-brown desert soil, gray-desert soil, and gray-calcium soil are dispersed in order, and saline soils are present in the lake basin area. Soil types have clear zonal features. The predominant vegetation types are those of the desert, the oasis, and the mountains. The Ejin Oasis region is home to natural poplar woods, and the Helan Mountains’ presence enhances the biodiversity and complexity of the Alxa Plateau system. One of the regions in China where sandstorms first appeared is the ecologically vulnerable Alxa region. Recent years have seen extensive artificial afforestation in the area surrounding the three main deserts. Saxaul, a genus of Quinoa, the genus has about 11 species, most of which are distributed in Central Asia, and there are two species in China (one species in Alxa League, and the other is the white pike, which is produced in the northern part of Xinjiang). Saxaul plantations in China’s western Inner Mongolia, Gansu, Qinghai and Xinjiang desert areas are distributed, born in the gravel desert, soil desert and desert. Saxaul plantations hardy, drought-resistant, saline and sand resistance, not only can curb land desertification, soil improvement, restoration of vegetation, but also to make the surrounding sandy grassland protection, in maintaining ecological balance on the maintenance of other species of trees plays an incomparable role, is the temperate desert in the important sand-fixing plants. Artificial Saxaul plantations are now a crucial man-made barrier to stop land desertification and encourage ecological regeneration in the area. **Figure 1** depicts the study area’s position schematically.

### 2.2 Experimental design

Plantations have been planted in the Alxa region since 1975. The study site for this experiment was a group of Saxaul plantations located near the southernmost boundary of the Badanjilin Desert in Alxa League. The site preserves a complete set of planting years since afforestation and is a typical area to study the effect of stand age gradient on soil water content and soil carbon density.2021 In August, using a spatial instead of temporal approach, the Saxaul plantations were gradually selected for the study in 2016, 2010, 1999, and 1975 (5yrs, 11yrs, 22yrs, and 46yrs later), and the nearby mobile sands (MS) as a control. 230 trees per hectare was the most widely used planting density in the study area. To ensure that the study was not influenced by planting density, 230 trees per hectare were selected. Three 5 m x 5 m plots were selected for sampling at each stand age, for a total of 15 plots at all stand ages. All ages of Saxaul plantations had the same soil texture, vegetation characteristics and precipitation at the time of planting. **Table 1** provides information on the sampling locations for each age class.

**Table 1:** The basic description of the studied sites.

Stand Age (a)		MS	5yrs	11yrs	22yrs	46yrs
Altitude (m)		1203.97	1204.86	1204.95	1203.69	1204.37
Clay Volume Fraction (%)		10.31	11.07	11.61	11.73	10.97
Silt Volume Fraction (%)		15.18	13.57	15.95	14.26	12.8
Sand Volume Fraction (%)		74.51	75.36	72.44	74.01	76.23
Diameter at breast height (cm)		/	3.5 ± 1.2	8.9 ± 3.8	12.4 ± 5.5	22.1 ± 7.5
Mean tree height (cm)		/	55 ± 12	140 ± 28	220 ± 46	420 ± 81
Planting density (Tree/hm^2^)		/	230	230	230	230
Crown width(cm)		/	59.3 ± 0.8	176.3 ± 1.5	185.7 ± 6.0	346.6 ± 5.7
Bulk Density (g cm^-3^)	0-100cm	1.49	1.49	1.51	1.52	1.52
	100-200cm	1.49	1.52	1.52	1.52	1.53
	200-300cm	1.5	1.51	1.52	1.53	1.53
pH	0 ~ 100cm	7.97	7.31	8.15	8.07	8.12
	100 ~ 200cm	8.01	8.43	8.03	8.03	8.21
	200 ~ 300cm	8.32	8.66	8.266	8.268	8.2

### 2.3 Soil collection and indoor analysis

**Figure 2:**
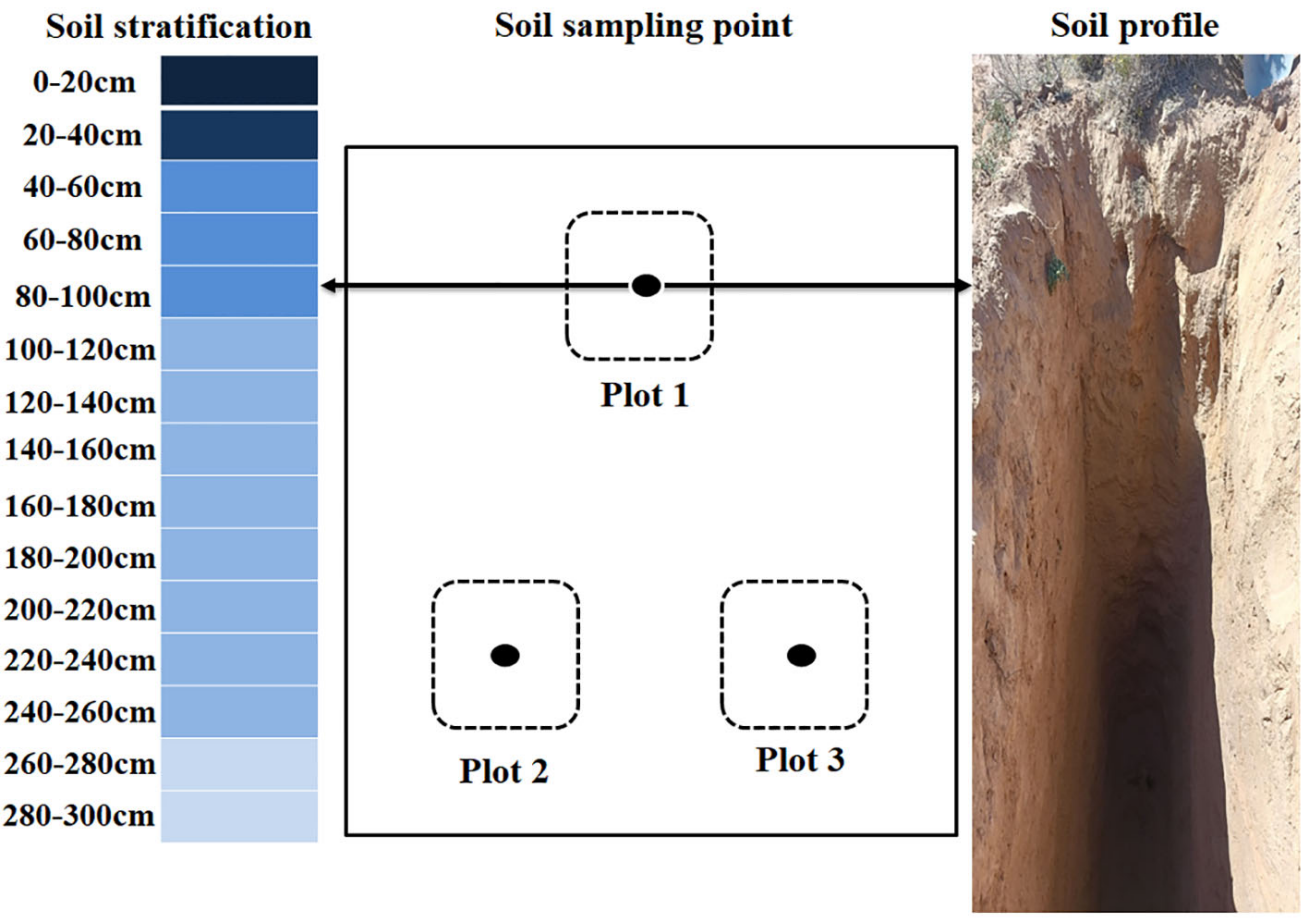
Schematic diagram of soil sampling.

In this study, the same soil stratum was sampled in three duplicates at each sample site in 20 cm strata. As shown in **Figure 2**. First, we chose three sample plots with about the same plant community composition within each of the five sample sites of MS, 5yrs, 11yrs, 22yrs, and 46yrs. Within the three sample locations of the five forest age sample plots, the soil obtained in each stratum was properly mixed before being split into two groups of samples. One group detects SWC, while the other group measures SIC and SOC content. Using a stainless steel soil sampler with a volume of 100 cm^3^, we gathered complete soil samples in order to calculate the soil volume. After collecting the soil, we air-dried it and passed it through a 2 mm sieve to remove any rocks or root systems larger than 2 mm. To analyze these samples, we ground them with a ball mill and passed them through a 100-mesh soil sieve. SOC content was measured by the K_2_Cr_2_O_7_–H_2_SO_4_ oxidation method, SIC content was determined by a modified pressure transducer method, soil pH (water to soil ratio of 2.5:1) was determined by the electrode method, and soil bulk weight was the dry mass per unit volume of soil at 105°C. The method for determining soil moisture content is the drying and weighing method. Under the condition of knowing the weight of the aluminum box, the fresh weight of the soil is first weighed, and then the soil sample is placed in an oven at 105-110 °C until a constant weight is reached. The lost mass is the moisture mass, which can be used to calculate the percentage of soil moisture. Soil fractions were determined by the gravimeter method using a 0.5 mol L^-1^ solution of sodium hexametaphosphate as dispersant and a Mastersizer 2000 particle size analyzer (Malvern Instruments Ltd., Worcestershire, UK). Percentage of soil particles for clay (less than 0.002 mm), chalk (0.002-0.05 mm), and sand (0.05-2 mm) were calculated based on the U.S. Soil Taxonomy.

### 2.4 Data analysis

SIC or SOC density(kg m^-2^) were calculated from soil bulk weight and inorganic or organic carbon content according to the following equation:


(1) $$SIC(SOC)density=SIC_{i}(SOC_{i})\times BD_{i}\times D_{i}/100$$

(2) $$SIC(SOC)storage=\sum_{i=1}^{n}SIC(SOC)dendity$$

where SIC_i_ (g·kg^-1^) is the SIC contents of the i-cm soil profile, SOC_i_ (g·kg^-1^) is the SOC contents of the i-cm soil profile, BD_i_ (g·cm^-3^) is the soil bulk density of the i-cm soil profile, and D_i_ (cm) is the soil layer thickness.

To assess how SWC, SIC, and SOC change with stand age, we determined the soil water deficit (SWD) and the SIC or SOC sequestration effect (SICSE or SOCSE) in soils at different stand age stages relative to the control value MS. SWD, SICSE, and SOCSE were calculated as follows:


(3) $$SWD_{j,k}=\frac{SWC_{j,k}-SWC_{0,k}}{SWC_{0,k}}$$

(4) $$SICSE{\left(SOCSE\right)}_{j,k}=\frac{SICD{\left(SOCD\right)}_{j,k}-SICD{\left(SOCD\right)}_{0,k}}{SICD{\left(SOCD\right)}_{0,k}}$$

where SWD_j,k_ is the SWD of soil layer k at stand j, SWC_j,k_ and SWC_0,k_ are the SWC (%) of soil layer k and control layer at stand j, respectively. SICSE_j,k_ and SOCSE_j,k_ are the SICSE and SOCSE of soil layer k at stand j. SICD_j,k_ and SICD_0,k_ are the SIC density (kg m^-2^) of soil layer k and control layer at stand j, respectively. SOCD_j,k_ and SOCD_0,k_ are the SOC densities (kg m^-2^) of layer k and control layer at stand age j, respectively.

All statistical analyses were performed using Origin 2022 and SPSS 23.0. One-way ANOVA followed by the Tukey’s HSD test (P<0.05) was used to compare the effects of vegetation restoration on SIC and SOC in the study sites.

## 3 Results

### 3.1 Profile variation of soil carbon content

**Figure 3:**
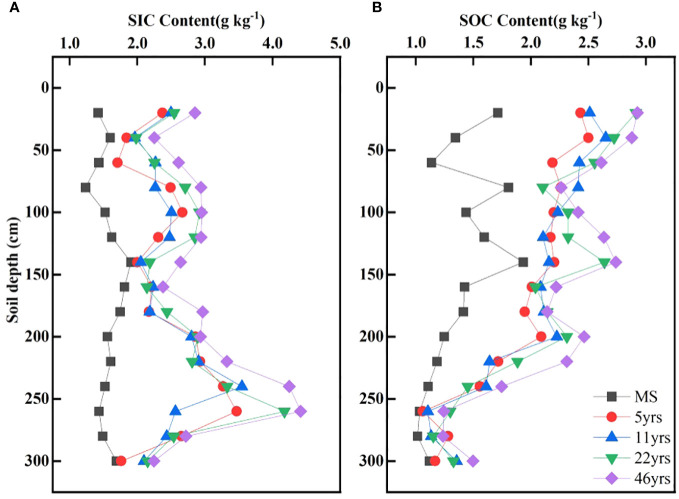
Variation of SOC and SIC along the soil depth. **(A, B)** characterize the variation of SIC and SOC with soil depth, respectively.

**Figure 3A** illustrates how the SIC content of various soil layers rose as stand age increased. After the Saxaul forest was planted, the soil profile’s overall SIC content was higher than it was for MS. The distribution of SIC content in the various soil layers revealed an overall trend of growing initially and then decreasing, with the deep layer at 250 cm exhibiting the highest value of SIC content. The SOC concentration of the soil profile was higher than that of MS, as shown in **Figure 3B**. As the age of the stand increased, so did the SOC content of the various soil layers. The vertical profile of the SOC content revealed a pattern of steadily declining SOC content. In contrast to the maximum value of SIC content, the maximum value of SOC content appeared at 20 cm of the soil surface layer as a result of litter. SIC and SOC levels varied less between different stand ages in the deeper soil layers.

The statistics of SIC and SOC contents at different forest age stages showed (**Tables 2**, **3**) that the mean values of SIC and SOC contents increased sequentially with increasing stand age. The SIC content varied in the order of 46yrs(2.96 g kg^-1^)>22yrs(2.66 g kg^-1^)>11yrs(2.45 g kg^-1^)=5yrs(2.45 g kg^-1^)>MS(1.57 g kg^-1^). SIC content varied from 1.23 - 1.91 g kg^-1^,1.71 - 3.47 g kg^-1^,1.96 - 3.55 g kg^-1^,1.98 - 4.17 g kg^-1^ and 2.25 - 4.41 g kg^-1^. The SOC content varied in the order of 46yrs(2.22 g kg^-1^)>22yrs(2.08 g kg^-1^)>11yrs(1.98 g kg^-1^)>5yrs(1.92 g kg^-1^)>MS(1.37 g kg^-1^). SOC content varied from 1.01 - 1.93 g kg^-1^,1.06 - 2.50 g kg^-1^,1.10 - 2.65 g kg^-1^,1.15 - 2.91 g kg^-1^ and 1.24 - 2.93 g kg^-1^.

**Table 2:** Descriptive statistics of SIC Content at 0 - 300 cm depth.

Planting Age	Min (g kg^-1^)	Max (g kg^-1^)	Mean (g kg^-1^)	SD (g kg^-1^)	CV (%)
MS	1.23	1.91	1.57	0.17	10.83
5yrs	1.71	3.47	2.45	0.53	21.68
11yrs	1.96	3.55	2.45	0.40	16.36
22yrs	1.98	4.17	2.66	0.56	21.01
46yrs	2.25	4.41	2.96	0.63	21.25

**Table 3:** Descriptive statistics of SOC Content at 0 - 300 cm depth.

Planting Age	Min (g kg^-1^)	Max (g kg^-1^)	Mean (g kg^-1^)	SD (g kg^-1^)	CV (%)
MS	1.01	1.93	1.37	0.29	21.09
5yrs	1.06	2.50	1.92	0.46	23.86
11yrs	1.10	2.65	1.98	0.50	25.01
22yrs	1.15	2.91	2.08	0.55	26.66
46yrs	1.24	2.93	2.22	0.55	24.98

### 3.2 Changes in soil carbon density and sequestration effects with stand age

**Figure 4:**
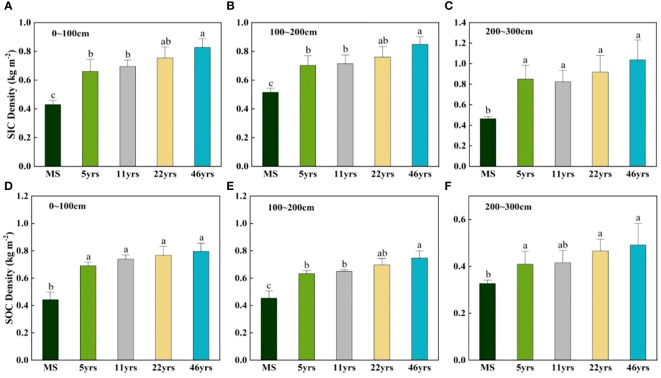
Changes in soil carbon density with stand age in different soil layers. Different lowercase letters indicate significant differences in SIC and SOC between different stand ages in the same soil layer (p< 0.05). **(A–C)** characterize the variation of SIC density in different soil layers for different stand ages. **(D–F)** show the variation characteristics of SOC density in different soil layers for different forest ages.

**Figure 5:**
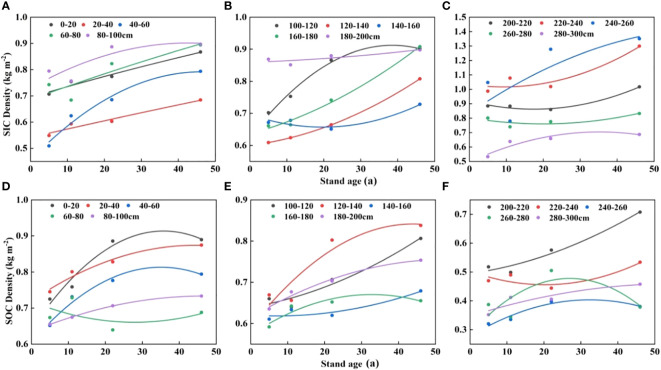
Relationship between stand age and soil carbon density. **(A–C)** show the variation characteristics of SIC density with stand age at different depths. **(D–F)** show the variation characteristics of SOC density at different depths with forest age.

As depicted in **Figure 4**, the SIC and SOC densities increased sequentially with the aging of the Saxaul stand in the 0-300 cm soil layer. After Saxaul was planted in sandy soils, the density of SIC and SOC was considerably higher (P<0.05) than MS at various stand age stages. While the SOC density did not substantially differ among stand ages, the SIC density in the 0-100 cm soil layer was significantly higher in stands older than 46 years (0.827 kg m^–2^) than those older than 11 years (0.695 kg m^–2^) and 5 years (0.660 kg m^–2^). The SIC and SOC density distribution features at various stand ages in the 100-200 cm soil layer were comparable to those in the 0 to 100 cm soil layer. At any stand age, the SIC and SOC densities in the 200-300 cm soil layer were not substantially different from one another and were only significantly different from the control MS. After Saxaul forests were planted in sandy areas, there was an overall noticeable increase in soil carbon density. Various soil strata had various increases in soil carbon density in terms of size. As can be seen in **Figure 5**, overall, both SOC and SIC densities showed an increasing trend with stand age, but not a significant linear relationship.

**Figure 6:**
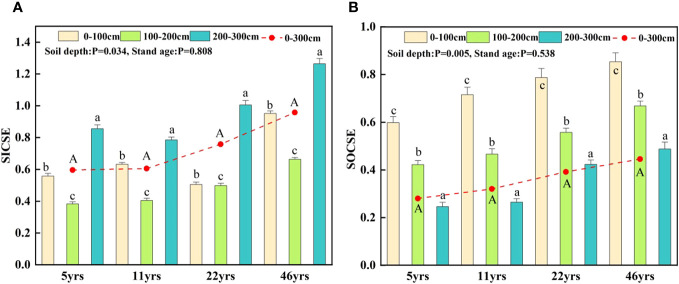
Characteristics of soil carbon sequestration effect with stand age. Different capital letters indicate significant differences in SICSE and SOCSE between different stand ages (p< 0.05), and different lower case letters indicate significant differences in SICSE and SOCSE between each 100 cm soil layer at the same stand age (p< 0.05). **(A)** shows the characteristics of SICSE with stand age for different soil depths. **(B)** shows the variation characteristics of SOCSE with forest age for different soil depths.

As demonstrated in **Figure 6**, SICSE and SOCSE increased as stand age increased. While SICSE and SOCSE varied considerably (P<0.05) at various soil depths within the same stand age, they did not differ significantly (P<0.05) with stand age. The mean SICSE values across the 0-300 cm soil profile were, in descending order, 46yrs (0.96), 22yrs (0.76), 11yrs (0.61), and 5yrs (0.59). The mean SOCSE values were in the following order: 46 years (0.67), 22 years (0.59), 11 years (0.48), and 5 years (0.42).

### 3.3 Effects of stand age on SWC and SWD

**Figure 7:**
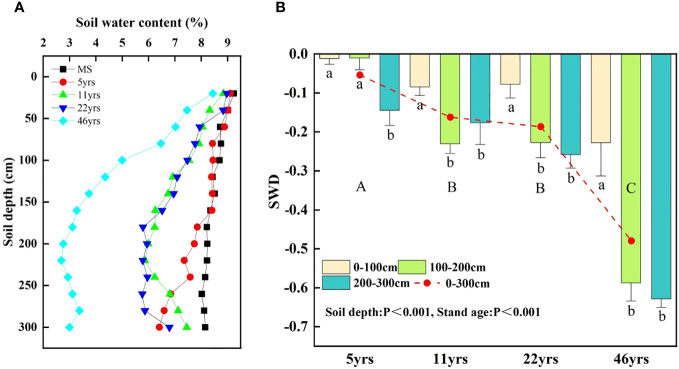
The vertical distribution of SWC and the variation characteristics of SWD in different forest ages, with different capital letters indicating significant differences in SWD between forest ages (p< 0.001) and different lowercase letters indicating significant differences between different soil layers of the same forest age (p< 0.05). **(A)** shows the variation characteristics of SWC with depth for different stand ages. **(B)** shows the variation characteristics of SWD with depth for different depths.

As the stand age increases, it is evident from **Figure 7A** that the profile SWC continually diminishes. The vulnerability of the top soil to recharge by precipitation may be the cause of the slight variation in SWC in the top 0-40 cm of soil. With increasing soil depth, the SWC disparity grew. The average SWC values in the 0-300 cm soil layer were MS (8.45%), followed in order by 5yrs (7.97%), 11yrs (7.08%), 22yrs (6.89%), and 46yrs (4.45%). Different stand age stages showed a substantially different mean SWC (p<0.05). For all stand ages, the mean SWC among them was 5.68%, which is 47.3% less than the control MS. The range of SWC variation was 8.02-9.24%, 6.41-9.11%, 5.85-8.84%, 5.76-8.96%, and 2.69-8.44% for MS, 5yrs, 11yrs, 22yrs, and 46yrs, respectively. As can be seen in **Figure 7B**, in the 0-300 cm soil layer, there was a significant trend of decreasing SWD with increasing stand age and soil depth (p< 0.001). The SWD was -0.058, -0.164, -0.188 and -0.481 for 5yrs, 11yrs, 22yrs and 46yrs, respectively (p< 0.05). At the same stand age, the SWD of deeper soils differed significantly (p< 0.05) than that of shallow soils.

**Figure 8:**
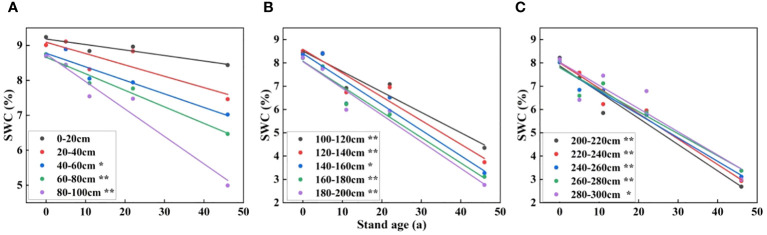
Relationship between stand age and soil moisture. **(A–C)** denote soil depths of 0-100 cm, 100-200 cm, and 200-300 cm, respectively. * denotes significance of linear regression at the 0.05 level. ** indicates significance of linear regression at the 0.01 level.

As can be seen from **Figure 8**, SWC showed a decreasing trend with increasing stand age. The decrease in SWC in the 0-40 cm soil layer with stand age gradient was not significant, and the rest of the soils showed significance. Shallow soils were not significant, probably because they were recharged by precipitation, while deeper soils were less affected by precipitation recharge, so the effect of stand age on SWC was more significant.

### 3.4 Relationship between soil moisture and soil carbon sequestration

**Figure 9:**
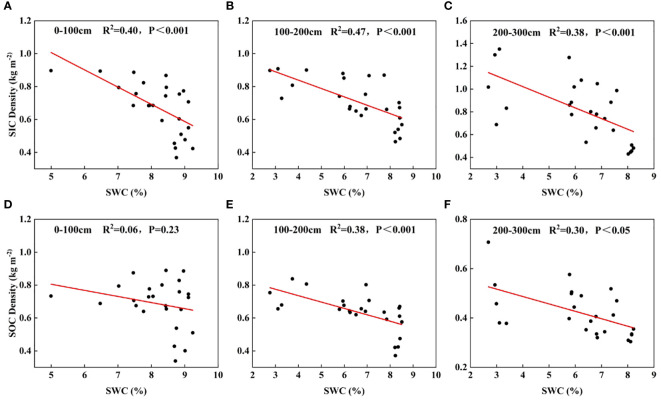
Relationship between soil moisture and soil carbon sequestration. **(A–C)** denote the relationship between SIC density and SWC at 0-100cm, 100-200cm, and 200-300cm soil depths, respectively; **(D–F)** denote the relationship between SOC density and SWC at 0-100cm, 100-200cm, and 200-300cm soil depths, respectively.

As can be seen from **Figure 9**, in this study, SIC density and soil moisture content were both significantly negatively correlated in 0-300 cm (P< 0.001). In the 0-100 cm soil layer, there was no significant correlation between SOC density and soil moisture, while in the 100-300 cm deep soil layer there was a negative correlation of different degrees.

## 4 Discussions

### 4.1 Effect of SWC on soil carbon sequestration

SWC is one of the fundamental factors that limit plant growth and development, and it is required for plant root growth, root respiration, and soil microbial activity. Large-scale afforestation continually depletes soil water, especially in arid and water-scarce locations, having a major impact on the coupling mechanism of soil carbon sequestration and soil water (Zhang and Shangguan, 2016). SIC had a significantly substantial negative connection with SWC in the 0-300 cm soil layer (**Figures 9A–C**) in this study. This finding is consistent with the Loess Plateau study (Zhao et al., 2016a; Lan et al., 2021). This is due to increased microbial activity in the soil as a result of enhanced rooting, which supports a rise in the partial pressure of CO_2_ in the soil, allowing for the partial breakdown of soil-forming carbonates in the topsoil. The migration of dissolved soil-forming carbonates to deeper soils and recrystallization at relatively low soil water contents resulted in a substantial negative connection between SIC content and SWC in the soil profile. There was no significant link between SOC and SWC in the 0-100 cm soil layer, but varied degrees of negative correlation were seen in the deeper 100-300 cm soils (**Figures 9D–F**), which is consistent with Zhang’s findings (Zhang and Shangguan, 2016). According to Chen Yongle’s study on soil carbon sequestration in various plantation forests in the Shapotou region, there was no discernible relationship between SWC and SOC in the shallow layers (0-100 cm) but a highly significant negative relationship between them in the 0-300 cm profile and the deeper layers (100-300 cm), respectively (Chen et al., 2018). The main sources of SOC are sand-fixing plant roots and secretions, and the decline in SWC in this layer also slows down further organic carbon breakdown. This phenomenon may be explained by the fact that soil microbial and animal activity is also relatively weak in deeper soils. SWC generally promotes or inhibits SOC sequestration through influencing plant root development, making its contribution to SOC variations particularly significant.

Since shallow SWC could be replenished by precipitation after plant growth was exhausted and SOC could also be successfully accumulated with input from root litter and microbial decomposition activities, the correlation between shallow SWC and SOC in this study was not statistically significant. Additionally, SWC, SOC, and SIC all displayed strong negative relationships in the deep soil. This is because, despite the well-developed root system of the forest floor having a great capacity to sequester carbon after afforestation of arid sandy soil, doing so typically requires more water (Zhao et al., 2007; Zhang and Shangguan, 2016). More water is needed for deeper soil carbon sequestration, and more carbon sequestration results in a greater loss of soil water. A deficiency in deep soil water brought on by long-term vegetation growth results in a dysregulation of the carbon-water coupling in the soil. The growth of vegetation’s fine roots and the entry of organic carbon into deep soil could both be hampered by a lack of water in the soil. Additionally, large-scale afforestation on the semi-arid and dry loess plateau resulted in soil water depletion, which in turn reduced the interaction between soil carbon and water (Wang et al., 2010a). In the arid and semi-arid loess plateau region, siding cypress monoculture planting can maintain relatively high soil water, but it has a low capacity to sequester carbon; in contrast, hybridizing acacia and buckthorn increases the soil carbon content, but severely depletes the SWC and causes severe drying of the soil (Li et al., 2021). According to this study, afforestation marginally enhanced soil carbon stocks, but it also significantly reduced soil water in the deep soil profile (**Figure 7**), causing the soil to dry up and become unsustainable. After sandy land was forested, the long-term cumulative evolution of soil carbon and water coupling may have undergone short-term cyclic alterations, which could explain the negative link between soil water and soil carbon sequestration. Afforestation promotes the development of sandy soils, resulting in the formation of loamy soils, which are formed at the expense of soil water depletion and soil drying, thus negatively affecting soil carbon sequestration (Zhao et al., 2007). Since soil water content and soil carbon sequestration are complementary processes, it can be argued that carbon accumulation and water depletion during vegetation growth are not two distinct processes. On the contrary, it can be argued that the increase in soil carbon stock after afforestation of sandy areas comes at the cost of soil water depletion.

### 4.2 Effect of afforestation on soil carbon sequestration

One crucial method for carrying out the ecological restoration of sandy or desertified soil is afforestation. In-depth research has been done on the impact of afforestation on soil carbon sequestration (Gao et al., 2018b; Shang et al., 2018). We also have a good time series to examine how soil carbon density varied with stand age in the Alxa region due to the installation of artificial plants on shifting sands at various dates. According to this study’s findings (**Figure 5**, not significant), SIC and SOC densities generally tend to rise as stand age rises. Numerous research (Guo and Gifford, 2002; Barcena et al., 2014; Wang et al., 2014) have shown that stand metabolism is one of the major factors influencing soil carbon changes. (Zhao et al., 2016a) in the Hunsandak sands similarly shown that soil carbon density increased with increasing stand age in poplar plantations. Long-term investigations by (Deng et al., 2014) revealed that soil carbon sequestration increased noticeably with recovery age. The planting of sorrel sand-fixing forests on the flowing dunes may have increased the amount and variety of above-ground plant life, which in turn reduced wind erosion and sand particle movement near the surface and improved the microenvironment of the sand surface. The carbon stock in the soil was further augmented by the carbon sequestration of soil microbial crusts that gradually developed on the top of sandy areas with increasing afforestation time (Su et al., 2010; Wang et al., 2010b). With increasing stand age in deeper soils, plant root density also increased, contributing to the sequestration and transformation of SOC and SIC. Soil carbon pools fluctuate once artificial forests are planted in sandy areas because vegetation alters the soil’s microclimate and soil structure.

Afforestation of sandy land increased soil carbon sequestration in this study, as both SIC and SOC rose with increasing stand age and the difference with the control MS was substantial. This outcome is in line with the rise in soil carbon stocks that followed the establishment of camphor pine, aspen, and Mongolian pine plantations in northwest China’s Mu Us Sands and Badain Jaran Desert (Su et al., 2010; Gao et al., 2018b). After establishing vegetation, due to the lower initial carbon density of flowing sandy soil, the levels of SOC and SIC significantly increase. Our results did not show that after afforestation, it is expected that the carbon in the soil will decrease due to the priming effect. The reason for this result may be that, firstly, in arid and semi-arid regions, the initial soil carbon content of mobile sandy land is relatively low; The second reason is that the soil moisture content is low, and the soil respiration rate is limited, leading to a decrease in the decomposition rate of soil carbon, resulting in long-term accumulation of soil carbon; Thirdly, our measurement year starts from the 5th year after planting the Saxaul, and the priming effect may occur exactly in the 1-5th year after planting the Saxaul, resulting in no observed reduction in soil carbon. According to this study, the soil carbon sequestration impact varied greatly depending on the soil layer but not significantly across different stand ages following sand afforestation. SICSE ranged from 200 to 300 cm at each stand age, followed by 0-100 cm and 100-200 cm (**Figure 6A**). The potential cause of this is that in deep soils with dry conditions, plant roots continue to grow downward and accumulate more root litter. This encourages soil microbial activity and speeds up the decomposition of root litter, which in turn causes the unstable SOC to be mineralized to produce more CO_2_ and further dissolve in the soil solution to form HCO_3_^-^, which then reacts with Ca_2_^+^ released from the decomposing litter to precipitate as CaCO_3_ (Zhao et al., 2016a). The mucilaginous sheath around the root hairs produced significant amounts of HCO_3_^-^ under respiration, making it a special environment for the combination of Ca_2_^+^ and HCO_3_^-^, which promoted the accumulation of secondary soil carbonate in the deep soil under both effects (Monger et al., 2015). In addition, the root system was enriched with excess Ca_2_^+^ and Mg_2_^+^ plasma. The majority of the carbon in deep soils in arid sandy plains comes from SIC. The largest SIC content in this study was in the soil layer below 200 cm, which was explained by studies on the distribution of SIC in various landscape types in temperate regions of northwest China, which revealed that more than 50% of SIC was situated at a depth of 100-300 cm (Wang et al., 2010a). In contrast, SOCSE showed a decreasing trend with soil depth, i.e., 0-100 cm > 100-200 cm > 200-300 cm (**Figure 6B**). On the one hand, it is caused by the continuous accumulation of organic matter due to biological processes of plant-soil interactions in shallow soils under increasing litter inputs (Zhang et al., 2015). On the other hand, after afforestation of sandy land, the top soil layer effectively reduces soil erosion under the action of tree apomictic cover, thus promoting the storage of soil organic carbon (Yang et al., 2022). This also explains the occurrence of a maximum SOC in the surface layer (0-20 cm). Additionally, as the soil profile deepens, the root density steadily declines, resulting in a reduction in the input, breakdown, and transformation of deep soil organic matter and a consequent continual decline in organic carbon sequestration. Additionally, this illustrates a highly substantial positive association between soil organic carbon and the root density of deep-rooted vegetation (Lan et al., 2021).

### 4.3 Effects of afforestation on soil water and insights

The foundation for vegetation restoration is moist soil, and vegetation has an impact on soil erosion and soil carbon sequestration. As a result, it is believed that soil water is crucial for soil carbon sequestration (Zhao et al., 2016b; Feng et al., 2017). In arid and semi-arid areas, mechanisms for soil carbon sequestration depend on soil water and are linked to the efficiency of soil water use (Lu et al., 2011). The deep SWC in this study drops more sharply, especially when the water table is deeper and the precipitation recharge is insufficient. The profile SWC in this study falls constantly with increasing HA plantations stand age. Stand age and soil depth have a big impact on soil water decrease during vegetation growth (Yang et al., 2022). Our findings demonstrated that SWC was strongly and adversely linked with stand age, meaning that SWC continued to decline as the stand age of the HA plantation increased. In contrast to the deep SWC reduction, the shallow SWC decrease has a short slope (**Figure 8**). Early on in the development of Saxaul plantations, there is less soil water loss and less soil desiccation due to the initial soil water being relatively abundant and the ability of artificial irrigation to promote pokeweed growth and development. Additionally, when the Saxaul forest expands and matures and its capacity for transpiration rises, the Saxaul plantations receive no additional artificial watering, which causes more severe soil desiccation and a more pronounced loss of soil water. This conclusion is supported by the findings of (Jia et al., 2017; Liu et al., 2018), who discovered a substantial positive association between stand age and stand transpiration water requirement in artificial vegetation on the Loess Plateau, which resulted in a constant decline in soil water. After vegetation building in the Loess Plateau, Yang et al., 2014 and Deng et al., (2016b) discovered that soil water continued to decline with increasing stand age.

On the SWD index, the impacts of soil depth and stand age were similarly quite significant (**Figure 7**). With increasing soil depth and stand age, the SWD index rose. Due to the retention of precipitation by leaves after the establishment of Saxaul forests in sandy areas, shallow soil water recharge is reduced to meet vegetation evapotranspiration losses. In order to maintain higher vegetation transpiration, the Saxaul forest root system uses deep soil water by hydraulic conduction, which exacerbates the decline in deep soil water content and soil water deficit (Eastham et al., 1990). In sandy environments, afforestation can minimize surface runoff and soil erosion while increasing the water-holding capacity of the soil. Although the increased soil water is unevenly distributed in time and space, this water provides a moist environment available for plant root uptake in the short term through soil infiltration. SWD and soil drying will restrict vegetation development as the age of the forest grows, resulting in vegetation degradation and ecological damage. Water stress may cause plantation trees to degrade and die more frequently as a result of projected future climate conditions that are dryer (Xin et al., 2008). Long-term soil carbon increases will be constrained as a result, and afforestation’s benefits as a carbon sink will be appropriately compensated. Future vegetation restoration efforts should concentrate more on carbon and water coordination, primarily to improve soil water resistance, in order to better preserve the stability of the ecosystem.

It is worth noting that the limitations of this study are mainly the lack of long-term continuous soil moisture monitoring data and the difficulty of quantifying potential soil moisture recovery. Nevertheless, soil moisture loss can be clearly observed in extensively planted Saxaul forests compared to mobile sandy areas. As the number of years of Saxaul planting increases, soil moisture loss in this area will further intensify. The Loess Plateau of China is another good example where irrational planting has created a layer of dry soil that adversely affects reforestation, which in turn threatens the health of the ecosystem in terms of carbon sequestration and soil and water conservation (Zhang and Shao, 2013). In order to make ecological construction sustainable, it is necessary to optimize the use of vegetation and to choose appropriate forest management practices rather than overemphasizing large-scale afforestation. In arid and semi-arid regions, large-scale afforestation has an important relationship with soil carbon sequestration capacity and soil moisture conservation. The coupling effect of carbon and water in arid regions is based on the rational utilization of water resources, which can better assess the sustainability of ecosystems (Zhang and Shangguan, 2016). In this study, soil water content was negatively correlated with soil carbon density, which also indicated that although afforestation improved wind-sand activities and accumulated soil carbon density to a certain extent, it came at the cost of depleting limited deep soil moisture. Deep soil carbon sequestration requires sufficient water, more carbon sequestration means more soil water consumption, and the lack of deep soil water leads to an imbalance in soil-carbon-water coupling. Afforestation on the Loess Plateau objectively reflects the fact that water stress leads to more frequent degradation and death of planted forests. Our study also shows that soil moisture is irreversibly lost with increasing stand age, and thus thinning measures are necessary to improve the efficiency of soil moisture utilization in arid regions for sustainable forest development.

## 5 Conclusion

The effects of afforestation of sandy areas in arid and semi-arid regions on soil water, soil carbon pools, and soil water-carbon were examined in this study. Large-scale “plantation” initiatives may have had a significant impact on soil water balance and soil carbon storage in northern China’s arid and semi-arid regions. The findings show that, whilst encouraging a modest increase in the soil carbon pool, afforestation of arid and semi-arid sandy areas also dramatically reduced deep soil water, leading to increasingly severe soil desiccation. This afforestation approach may not be viable in arid areas since the equal increase in soil carbon pool comes at the expense of water loss. Soil moisture will irreversibly lose with the increase of forest age, so it is necessary to adopt thinning measures to improve the utilization efficiency of soil moisture in arid areas and achieve sustainable development of forests.

## Data availability statement

The original contributions presented in the study are included in the article/supplementary material. Further inquiries can be directed to the corresponding author.

## Author contributions

XZ: Data curation, Visualization, Writing – original draft, Writing – review & editing. JS: Funding acquisition, Methodology, Resources, Supervision, Writing – review & editing. XH: Funding acquisition, Supervision, Writing – review & editing. BJ: Funding acquisition, Supervision, Writing – review & editing. DZ: Data curation, Investigation, Writing – review & editing. CW: Data curation, Investigation, Writing – review & editing. JQ: Investigation, Software, Writing – review & editing. ZL: Data curation, Investigation, Writing – review & editing.
